# Supplementary material for: Prevalence and characteristics of long COVID in elderly patients: An observational cohort study of over 2 million adults in the US
Source: PLoS Med. 2023 Apr 17;20(4):e1004194. doi: 10.1371/journal.pmed.1004194 (PMC10150975; doi:10.1371/journal.pmed.1004194)
Supplement: S2 Table — (DOCX) [file pmed.1004194.s003.docx]

S2 Table Exclusion of symptoms by comorbidities

|  | **Fatigue /malaise /weakness** | **muscle/ joint pain** | **dyspnea** | **cough** | **chest pain** | **palpitations** | **sleep disturbance** | **loss of taste /smell** | **headache** | **cognitive impairment (brain fog)** | **Memory problem** |
| --- | --- | --- | --- | --- | --- | --- | --- | --- | --- | --- | --- |
| Acute myocardial infarction |  |  | y |  | y |  |  |  |  |  |  |
| Atrial Fibrillation |  |  |  |  |  | y |  |  |  |  |  |
| Chronic obstructive pulmonary disease |  |  | y | y |  |  |  |  |  |  |  |
| Heart Failure |  |  | y |  |  |  |  |  |  |  |  |
| Ischemic Heart Disease |  |  |  |  | y |  |  |  |  |  |  |
| Depression |  |  |  |  |  |  | y |  |  |  |  |
| Alzheimer’s Disease or Dementia |  |  |  |  |  |  |  |  |  | y | y |
| Rheumatoid Arthritis/Osteoarthritis |  | y |  |  |  |  |  |  |  |  |  |
| Stroke/Transient Ischemic Attack |  |  |  |  |  |  |  |  |  | y | y |
| Asthma |  |  | y |  |  |  |  |  |  |  |  |
| Anxiety Disorders |  |  |  |  |  |  | y |  |  |  |  |
| Bipolar Disorder |  |  |  |  |  |  | y |  |  |  |  |
| Major Depressive Affective Disorder |  |  |  |  |  |  | y |  |  |  |  |
| Cystic Fibrosis |  |  | y |  |  |  |  |  |  |  |  |
| Fibromyalgia, Chronic Pain and Fatigue | y | y |  |  |  |  |  |  |  |  |  |
| Migraine And Other Chronic Headache |  |  |  |  |  |  |  |  | y |  |  |
