# Supplementary material for: Prevalence and characteristics of long COVID in elderly patients: An observational cohort study of over 2 million adults in the US
Source: PLoS Med. 2023 Apr 17;20(4):e1004194. doi: 10.1371/journal.pmed.1004194 (PMC10150975; doi:10.1371/journal.pmed.1004194)
Supplement: S3 Table — (DOCX) [file pmed.1004194.s004.docx]

S3 Table Patient characteristics in the 2018 and 2019 Flu seasons (IQR – inter-quartile range, LIS – low- income subsidy)

|  | **2018 Flu season** | **2019 Flu season** |
| --- | --- | --- |
| N (%) | 641709 (100) | 312137 (100) |
| Any prior hospitalization (-1 year to -2 weeks) | 150,905(23.5) | 75,424(24.2) |
| Overall death | 60,072(9.4) | 28,144(9.0) |
| Charlson comorbidity index; Median (IQR) | 2.0±2.3; 1.0(0.0-3.0) | 2.0±2.3; 1.0(0.0-3.0) |
| Age at influenza diagnosis, Median (IQR) | 75.0(70.0-83.0) | 75.0(70.0-82.0) |
| 65-69 | 135,361(21.1) | 66,092(21.2) |
| 70-74 | 164,578(25.6) | 85,785(27.5) |
| 75-79 | 123,044(19.2) | 61,999(19.9) |
| 80-84 | 90,938(14.2) | 44,123(14.1) |
| 85+ | 127,788(19.9) | 54,138(17.3) |
| Female | 383,318(59.7) | 188,203(60.3) |
| Race: White | 525,516(81.9) | 250,692(80.3) |
| Black | 43,786(6.8) | 22,173(7.1) |
| Hispanic | 37,828(5.9) | 20,862(6.7) |
| Asian | 18,610(2.9) | 9,394(3.0) |
| Other | 15,969(2.5) | 9,016(2.9) |
| Region: Northeast | 100,197(15.6) | 59,047(18.9) |
| Midwest | 139,108(21.7) | 56,501(18.1) |
| South | 291,370(45.4) | 141,934(45.5) |
| West | 103,038(16.1) | 49,570(15.9) |
| Income: Ever Dual | 128,141(20.0) | 63,141(20.2) |
| Non-Dual LIS | 11,380(1.8) | 6,572(2.1) |
| Non-Dual Non-LIS | 502,188(78.3) | 242,424(77.7) |
