# Supplementary material for: Prevalence and characteristics of long COVID in elderly patients: An observational cohort study of over 2 million adults in the US
Source: PLoS Med. 2023 Apr 17;20(4):e1004194. doi: 10.1371/journal.pmed.1004194 (PMC10150975; doi:10.1371/journal.pmed.1004194)
Supplement: S4 Table — (DOCX) [file pmed.1004194.s005.docx]

S4 Table COVID-19 outpatient cases and controls (SD – standard deviation, IQR – inter-quartile range, LIS – low-income subsidy)

|  | **Outpatient Covid** | **1:5 Matched Non-Covid Control** | **P-value** | **Standardized Difference†** |
| --- | --- | --- | --- | --- |
| N | 1,479,183 | 6,286,633 |  |  |
| Any prior hospitalization (-1 year to -2 weeks) | 349,688(23.6) | 1,064,736(16.9) | <.0001 | 0.174 |
| Overall death | 232,844(15.7) | 416,835(6.6) | <.0001 | 0.332 |
| Charlson comorbidity index; Mean±SD | 1.9±2.3 | 1.9±2.1 | <.0001 | 0.008 |
| Age at diagnosis; Mean±SD | 77.0±8.2 | 77.2±8.1 | <.0001 | -0.032 |
| 65-69 | 313,613(21.2) | 1,203,264(19.1) | <.0001 | 0.052 |
| 70-74 | 389,872(26.4) | 1,656,204(26.3) | 0.758 | 0.000 |
| 75-79 | 277,075(18.7) | 1,231,928(19.6) | <.0001 | -0.022 |
| 80-84 | 205,435(13.9) | 927,642(14.8) | <.0001 | -0.025 |
| 85+ | 293,188(19.8) | 1,267,595(20.2) | <.0001 | -0.009 |
| Female | 853,157(57.7) | 3,691,526(58.7) | <.0001 | -0.021 |
| Race: White | 1,165,494(78.8) | 5,082,215(80.8) | <.0001 | -0.052 |
| Black | 117,273(7.9) | 458,930(7.3) | <.0001 | 0.024 |
| Hispanic | 111,488(7.5) | 411,180(6.5) | <.0001 | 0.040 |
| Asian | 37,698(2.5) | 149,087(2.4) | <.0001 | 0.012 |
| Other | 47,230(3.2) | 185,221(2.9) | <.0001 | 0.014 |
| Region: Northeast | 322,838(21.8) | 1,335,589(21.2) | <.0001 | 0.014 |
| Midwest | 309,689(20.9) | 1,339,240(21.3) | <.0001 | -0.009 |
| South | 566,699(38.3) | 2,443,102(38.9) | <.0001 | -0.011 |
| West | 265,878(18.0) | 1,126,235(17.9) | 0.0875 | 0.002 |
| Income: Ever Dual | 367,459(24.8) | 1,478,873(23.5) | <.0001 | 0.031 |
| Non-Dual LIS | 21,666(1.5) | 83,000(1.3) | <.0001 | 0.013 |
| Non-Dual Non-LIS | 1,090,058(73.7) | 4,724,760(75.2) | <.0001 | -0.034 |

† Standardized difference = difference in means or proportions divided by standard error
